# Long-term results following off-pump coronary-artery bypass grafting in left ventricular dysfunction

**DOI:** 10.1007/s00380-024-02383-9

**Published:** 2024-03-10

**Authors:** Masahiro Ikeda, Hiroshi Niinami, Kozo Morita, Satoshi Saito, Akihiro Yoshitake

**Affiliations:** 1 Department of Cardiovascular Surgery, Tokyo Women’s Medical University Hospital, 8-1, Kawada-Cho, Shinjuku-Ku, Tokyo, 162-8666 Japan; 2 Department of Cardiovascular Surgery, Saitama Medical University International Medical Center, Saitama, Japan

**Keywords:** Off-pump coronary artery grafting (OPCAB), Bilateral internal thoracic arteries (BITA), Severe left ventricular (LV) dysfunction, Low LV ejection fraction

## Abstract

Severe left ventricular (LV) dysfunction is an independent risk factor for early and long-term mortality after coronary-artery bypass grafting (CABG). Off-pump CABG (OPCAB) significantly reduces the early incidence of major complications in high-risk patients. Moreover, bilateral internal thoracic artery (BITA) grafting after CABG is associated with improved long-term outcomes. We aimed to evaluate the impact of multivessel OPCAB with BITA grafting for complete revascularization on postoperative and long-term outcomes in patients with low LV ejection fraction (EF). We included 121 patients with EF ≤ 30.0% who underwent isolated multivessel OPCAB (average LVEF, 24.8%) between April 2007 and December 2019. Sixty-six patients received BITA grafts, while 55 had single internal thoracic artery (SITA) grafts. We conducted multivariate analyses to examine the correlation between perioperative data and late mortality rate. The early mortality rate was 1.65%. After excluding in-hospital mortality cases, we performed long-term follow-up of 119 patients. Early postoperative echocardiography showed significant LVEF improvement in 89 (75.2%) patients. However, LVEF remained ≤ 30.0% in 30 (24.8%) patients. We recorded 15 and 30 cases of cardiac death and cardiac events, respectively, during the long-term follow-up period. Postoperative LVEF ≤ 30.0% (*P* < 0.01) and no use of BITA grafting (*P* = 0.03) were significant predictors of cardiac death and events; moreover, hemodialysis was a significant predictor of all-cause mortality rather than cardiac death. Multivessel OPCAB in patients with severe LV dysfunction was associated with acceptable in-hospital mortality and early postoperative improvement in LV function. Additionally, OPCAB with BITA grafting may provide long-term benefits with respect to cardiac death and events. However, the long-term benefits were significantly limited in patients without early postoperative improvement in LV function and patients with chronic hemodialysis.

Clinical registration number: 5590 (14/5/2020 Tokyo Women’s Medical University).

## Introduction

Ischemic heart disease with severe left ventricular (LV) dysfunction is reportedly an independent risk factor for early and long-term postoperative mortality [1–4]. Several non-randomized studies and the Surgical Treatment for Ischemic Heart Failure (STICH) randomized trial have consistently reported that coronary artery bypass grafting (CABG) improved survival rates and decreased cardiovascular mortality rates among patients with low LV ejection fraction (LVEF) [5, 6]. Off-pump CABG (OPCAB) in high-risk patients with severe LV dysfunction has been associated with a significant reduction in the incidence of early incidence of major complications [7–9]. Nonetheless, fewer distal anastomoses and incomplete revascularizations have been reported in OPCAB compared with conventional CABG using a cardiopulmonary pump (ONCAB), and these fewer anastomoses were considered inadequate for such patients with severe LV dysfunction [9, 10]. Therefore, the long-term advantage of OPCAB remains unclear.

Compared with single internal thoracic artery (SITA) grafting, bilateral internal thoracic artery (BITA) grafting provides improved clinical and long-term survival benefits in patients with normal LVEF [11–16]. Additionally, BITA grafting does not increase perioperative complications in high-risk patients [15, 16]. However, the low survival rate among patients with severe LV dysfunction has limited the long-term benefits of BITA grafting after undergoing CABG [16, 17].

We performed multivessel OPCAB using BITA for complete revascularization and long-term benefits. This study aimed to evaluate and compare the operative and long-term outcomes of OPCAB using BITA or SITA in patients with severe LV dysfunction (LVEF ≤ 30%).

## Materials and methods

### Study design

We retrospectively collected data from medical records, catheterization reports, angiograms, and echocardiograms. LV function was determined using transthoracic echocardiography based on the modified Simpson method. We reviewed the ventriculograms obtained during coronary angiography and magnetic resonance imaging (MRI) only when available. Severe LV dysfunction was defined as LVEF ≤ 30% in this study.

A total of 121 adult patients with LVEF ≤ 30% underwent isolated multivessel OPCAB at our institutions (107 patients in Saitama Medical University International Medical Center from April 2007 to June 2017, 14 patients in Tokyo Women’s Medical University from July 2017 to December 2019). We discussed treatment strategies and presented our OPCAB results to all patients and allowed them to select their preferred strategy. All patients provided consent to our strategies, and most of their operations were performed by the chief surgeon (HN) of our team, who discussed strategies and ideas at a heart team conference. In our experience at our institutions, most cardiologists who referred these patients and the patients themselves preferred OPCAB, with only a few patients choosing ONCAB.

We excluded patients with single-vessel disease and those scheduled for ONCAB for concomitant procedures. In contrast, we included patients who underwent intraoperative conversion to ONCAB for intraoperative fatal arrhythmia or hemodynamic breakdown, including hypotension or pulmonary hypertension. Finally, we analyzed 121 patients (55 and 66 with SITA and BITA grafts, respectively). The use of BITA or SITA grafts was selected at the discretion of the surgeon.

### Operative procedure

Preoperative prophylactic intra-aortic balloon pumping (IABP) was administered at the surgeon’s discretion. Complete myocardial revascularization using the OPCAB technique via a median sternotomy was planned for all surgeries. In case of failed management of intraoperative fatal arrhythmia or hemodynamic instability using inotropic agents or an IABP, we performed intraoperative conversion to ONCAB. We employed the beating CABG technique without a cross clamp during conversion to ONCAB [18–20]. All internal thoracic arteries (ITAs) were harvested in a complete skeletonized fashion using a harmonic scalpel to prevent sternal wound infection [21, 22]. We used the ITAs as in situ grafts whenever possible; at least one in situ ITA was anastomosed to the left anterior descending artery. For some cases, we removed the ITA and used it as a free graft for the in situ contralateral ITA (Y-graft). The ante-aortic crossover right ITA bypass graft was protected using a pedicle thymus flap [23]. Regarding other grafts, the saphenous vein graft (SVG) was the most commonly used, with the gastroepiploic artery and radial artery being also used.

All discharged patients underwent echocardiographic assessment within 3 postoperative months.

### Follow-up

After excluding two cases due to in-hospital mortality, we included 119 patients in the long-term analysis. Up-to-date clinical follow-up was performed through telephone interviews with the patients or their families. We recorded the occurrence of postoperative cardiac events, which were defined as any of the following complications: sudden death, recurrent angina, myocardial infarction, congestive heart failure requiring hospital readmission, percutaneous coronary intervention, or reoperation.

### Statistical analysis

The distribution of the baseline variables and perioperative events were presented as means ± standard deviation (SD) and proportions for continuous and categorical variables, respectively. Between-group comparisons were performed using the unpaired Student’s *t* test, *χ*^2^ test, or Fisher’s exact test, as appropriate. Binary logistic and Cox proportional hazards regression analyses were performed to identify predictive factors for long-term mortality and to assess the influence of perioperative characteristics on late outcomes. Additionally, we analyzed the predictive utility of BITA graft use and postoperative LVEF for long-term outcomes. All significant variables (*P* > 0.2) from the univariable analysis were entered into multivariate models using a stepwise backward elimination technique. Odds ratios or hazard ratios (HR) with 95% confidence intervals were determined for each variable. Moreover, we performed non-parametric estimates of freedom from cardiac death and events using the Kaplan–Meier method. Log-rank statistics were used to compare the survival curves of patients who underwent alternative revascularization techniques.

The log-rank test was used to compare survival curves. Statistical significance was set at *P* < 0.05. All statistical analyses were performed using the IBM SPSS software version 27 for Windows (IBM Software Group, Armonk, NY, USA).

## Results

### Patient characteristics

Table 1 summarizes the preoperative and operative characteristics of the included patients in this study. There were no significant between-group differences in preoperative characteristics, except for the proportion of octogenarians (SITA, 27.2%; BITA, 1.5%; *P* = 0.001) and chronic hemodialysis (HD) patients (SITA, 29.1%; BITA, 13.6%; *P* = 0.02). Additionally, there was no significant between-group difference in the mean LVEF (total, 24.8%; SITA, 24.7%; BITA, 24.8%; *P* = 0.87).

**Table 1:** Preoperative and operative characteristics: octogenarians and chronic hemodialysis cases were significant

	Total (*n* = 121)	SITA group (*n* = 55)	BITA group (*n* = 66)	*P* value
Preoperative characteristics				
Age, mean ± SD y.o	66.4 ± 10.9	69.8 ± 10.7	63.6 ± 10.3	0.0007
Octogenarians, *n* (%)	16(13.2)	15(27.2)	1(1.5)	0.001
Male, *n* (%)	95(75.2)	38(69.1)	53(80.3)	0.1
Hypertension, *n* (%)	84(69.4)	40(72.7)	44(66.7)	0.28
CKD on HD, *n* (%)	25(20.7)	16(29.1)	9(13.6)	0.02
Diabetes,* n* (%)	75(62.0)	33(60.0)	12(63.6)	0.22
Insulin dependent, *n* (%)	24(19.8)	9(16.4)	15(22.7)	0.19
COPD, *n* (%)	18(14.9)	10(18.2)	8(12.1)	0.23
CHF (NYHA ≥ 3), *n* (%)	98(81.0)	41(75.5)	57(86.4)	0.45
ACS, *n* (%)	17(14.1)	10(18.2)	7(10.6)	0.09
LVDd ± SD mm	57.8 ± 6.68	58.2 ± 6.68	59.7 ± 7.11	0.16
MR ≥ moderate, *n* (%)	15(12.4)	9(16.4)	6(9.0)	0.12
EF, mean ± SD %	24.8 ± 4.5	24.7 ± 4.15	24.8 ± 4.75	0.87
Operative characteristics				
Average distal anastomosis, Mean ± SD (range)	3.54 ± 0.94 (2–6)	3.38 ± 0.76 (2–5)	3.67 ± 1.06 (2–6)	0.1
Emergent operation, *n* (%)	24(19.8)	14(25.5)	10(15.2)	0.08
IABP, *n* (%)	85(70.3)	40(72.7)	45(68.2)	0.25
Conversion, *n* (%)	4(3.31)	2(3.64)	2(3.03)	0.43
Complete revascularization, *n* (%)	117(97.0)	52(94.5)	65(98.5)	0.12

*SD* standard deviation, *CKD* chronic kidney disease, *HD* hemodialysis, *COPD* chronic obstructive pulmonary disease, *CHF* congestive heart failure, *NYHA* New York Heart Association, *ACS* acute coronary syndrome, *LVDd* left ventricular end-diastolic diameter, *MR* mitral regurgitation, *EF* ejection fraction, *IABP* intra-aortic balloon pumping

### Operative characteristics

There were no significant between-group differences in operative characteristics (Table 1). The average number of distal anastomoses and rate of complete revascularization were 3.54 (SITA, 3.38 (2–5); BITA, 3.67 (2–6); *P* = 0.10) and 97.0% (SITA, 94.5%; BITA, 98.5%; *P* = 0.12), respectively. Sequential bypass was mainly used for cases in which the number of anastomoses exceeded that of grafts. The proportions of IABP support, emergent cases, and converted cases were 70.3% (SITA, 72.7%; BITA, 68.2%; *P* = 0.25), 19.8% (SITA, 25.5%; BITA, 15.2%; *P* = 0.08), and 3.31% (SITA, 3.64%; BITA, 3.03%; *P* = 0.43), respectively.

### Mortality and morbidities

There were two cases of in-hospital mortality in the SITA group. The causes of death were non-occlusive mesenteric ischemia from low output syndrome and ventricular tachycardia with reperfusion injury. Surgical site infections were observed in 5 cases (total, 4.10%; SITA, 5.46%; BITA, 3.03%; *P* = 0.26). Three cases were closed with direct re-suture or vacuum-assisted closure therapy without sternal reconstruction. Two cases were closed with muscle flap after controlled wound condition with vacuum-assisted closure therapy following sternal debridement as deep sternal wound infection (total, 1.65%; SITA, 1.82%; BITA, 1,52%; *P* = 0.89) [24]. In each case, infection was controlled within the local site and none were associated with mortality or other complications. There was no significant between-group difference in the in-hospital mortality rate (total, 1.65%; SITA, 3.64%; BITA, 0%; *P* = 0.08) or early postoperative complication rate (Table 2).

**Table 2:** Early mortality and morbidities

	Total	SITA group	BITA group	P value
	(*n* = 121)	(*n* = 55)	(*n* = 66)	
Hospital mortality, *n* (%)	2 (1.65)	2 (3.64)	0 (0)	0.08
Bleeding, *n* (%)	3 (2.46)	2 (3.64)	1 (1.52)	0.23
Permanent stroke, *n* (%)	0 (0)	0 (0)	0 (0)	NS
Prolonged ventilation, *n* (%)	25 (20.5)	14 (25.5)	11 (16.7)	0.08
PMI, *n* (%)	0 (0)	0 (0)	0 (0)	NS
ARF on temporary HD, *n* (%)	18 (14.8)	10 (18.2)	8 (12.1)	0.08
SSI, *n* (%)	5 (4.10)	3 (5.46)	2 (3.03)	0.26
DSWI, n (%)	2(1.65)	1(1.82)	1(1.52)	0.89

*Bleeding* postoperative bleeding requiring re-sternotomy, prolonged ventilation: more than 72 h postoperative intubation time, *PMI* perioperative myocardial infarction with increased CK-MB and angiographic occlusion of graft or native coronary, *ARF* acute renal failure requiring new temporary hemodialysis, *HD* hemodialysis, *SSI* surgical site infection, *DSWI* deep sternal wound infection

### Postoperative LV function

**Fig. 1:**
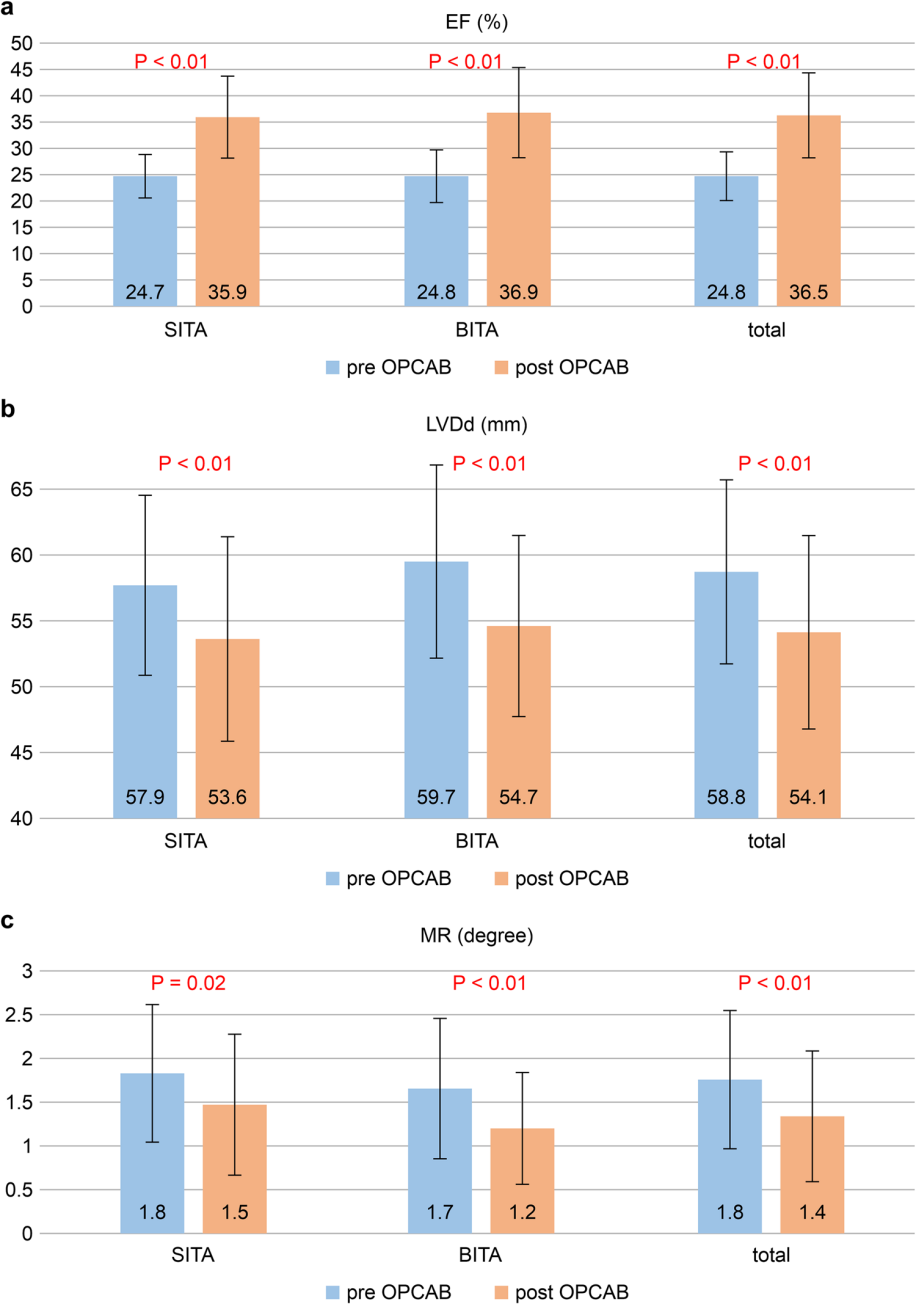
Echocardiogram within 3 months after surgery showed significant early improvement in LV function in both groups (**a** LVEF, **b** LVDd, **c** MR). *EF* ejection fraction, *SITA* single internal thoracic artery, *BITA* bilateral internal thoracic artery, *OPCAB* off-pump coronary artery bypass grafting, *LVDd* left ventricular end-diastolic diameter, *MR* mitral regurgitation, *LV* left ventricle

We analyzed the postoperative LV function within 3 postoperative months using echocardiography in all discharged patients. We observed significant early postoperative improvement in LV function in both groups, with an increase of LVEF > 10% (preoperative EF, 24.8%; postoperative EF, 36.5%; *P* < 0.01: Fig. 1a), LV remodeling (preoperative left ventricular end-diastolic diameter [LVDd], 58.8 mm; postoperative LVDd, 54.1 mm; *P* < 0.01: Fig. 1b), and mitral regurgitation (MR) improvement (preoperative degree of MR, 1.8; postoperative degree of MR, 1.4; *P* < 0.01: Fig. 1c). There were no significant between-group differences in the rates of these early improvements. However, LVEF remained at ≤ 30.0% in 30 patients with both SITA and BITA groups (total, 25.2%; SITA, 28.3%; BITA, 22.7%; *P* = 0.318).

### Long-term results

We analyzed the follow-up data of 119 patients who survived until hospital discharge. The mean follow-up period was 83.0 months (maximum 166.0 months). None of the patients were lost to follow-up. During the follow-up period, 15 cardiac deaths and 30 cardiac events occurred.

**Fig. 2:**
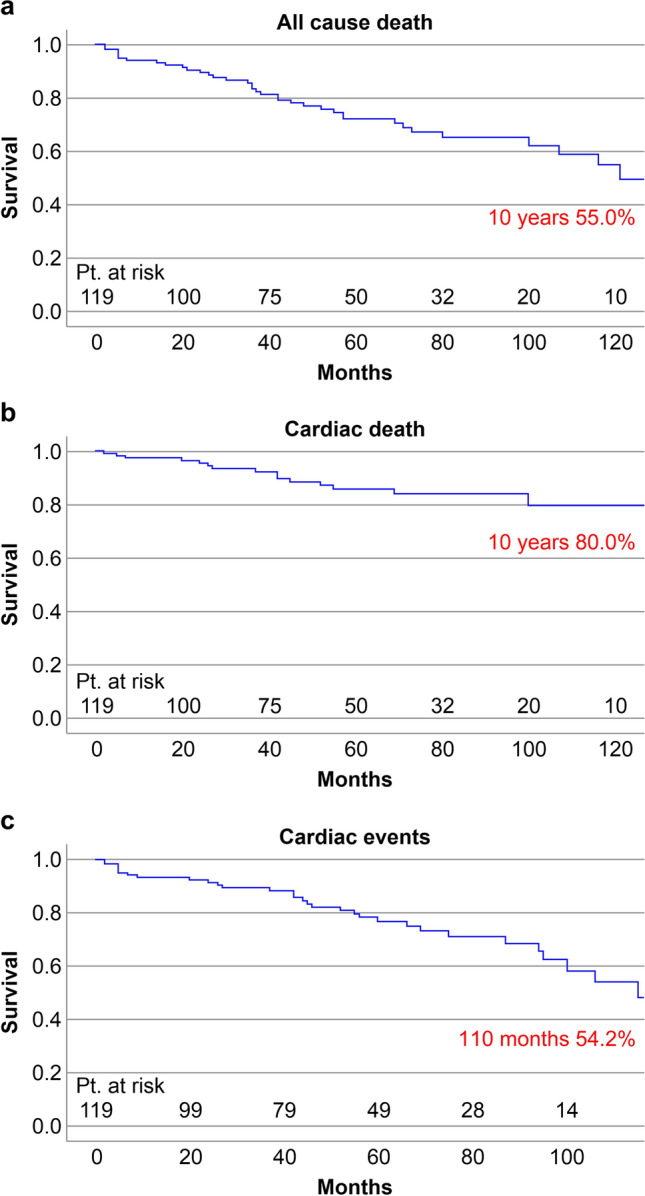
Survival analysis with Kaplan–Meier for freedom from all-cause death (**a**), cardiac death (**b**), and cardiac events (**c**)

Figure 2 outlines freedom from all-cause death (a), cardiac death (b), and cardiac events (c). The 10-year survival rate for cardiac death was 80.0%.

We used the Cox multivariate proportional hazards model to identify independent risk factors for all-cause death, late cardiac death, and cardiac events. Chronic kidney disease (CKD) on HD (*P* = 0.001, HR 3.346) and postoperative EF ≤ 30% (*P* = 0.012, HR 2.333) were significant predictors of all-cause death. Postoperative EF ≤ 30% and absence of BITA were significant predictors for late cardiac death (postoperative EF ≤ 30%: *P* = 0.001, HR 6.444; BITA: *P* = 0.017, HR 0.250) and cardiac events (postoperative EF ≤ 30%: *P* = 0.007, HR 2.758; BITA: *P* = 0.006, HR 0.333). Many octogenarians survived past the age of 90 years, with no significant between-group differences (Table 3).

**Table 3:** Univariable analysis and Cox regression analysis

	Univariable	Multivariable			
	*P* value	*P* value	HR	95% CI	
All-cause death					
Octogenarians	0.84				
Male	0.19				
Hypertension	0.52				
CKD on HD	0.001	0.001	3.346	1.682	6.626
Diabetes	0.42				
Insulin dependent	0.82				
COPD	0.04	0.12			
CHF (NYHA ≥ 3)	0.94				
ACS	0.28				
MR ≥ moderate	0.32				
BITA	0.01	0.07			
P-EF ≤ 30%	0.009	0.012	2.333	1.201	4.534
P-EF ≤ 35%	0.06				
Cardiac death					
Octogenarians	0.99				
Male	0.95				
Hypertension	0.99				
CKD on HD	0.70	0.878			
Diabetes	0.98				
Insulin dependent	0.88				
COPD	0.13				
CHF (NYHA ≥ 3)	0.052				
ACS	0.43				
MR ≥ moderate	0.92				
BITA	0.01	0.017	0.25	0.08	0.777
P-EF ≤ 30%	0.001	0.001	6.444	2.228	18.637
P-EF ≤ 35%	0.09				
Cardiac events					
Octogenarians	0.95				
Male	0.45				
Hypertension	0.87				
CKD on HD	0.26	0.77			
Diabetes	0.23				
Insulin dependent	0.37				
COPD	0.22				
CHF (NYHA ≥ 3)	0.07				
ACS	0.57				
MR ≥ moderate	0.82				
BITA	0.002	0.006	0.333	0.152	0.729
P-EF ≤ 30%	0.001	0.007	2.758	1.326	5.735
P-EF ≤ 35%	0.28				

Cox regression analysis: For all-cause death, CKD on HD (*P* = 0.001, HR 3.346) and postoperative EF ≤ 30% (*P* = 0.012, HR 2.333) were identified as the significant predictors. For late cardiac death and cardiac events, absence of BITA (BITA: *P* = 0.017, HR 0.25; *P* = 0.006, HR 0.33) and postoperative EF ≤ 30% (*P* = 0.001, HR 6.444; *P* = 0.007, HR 2.758) were identified as the significant predictors and CKD on HD was not identified as significant

*HR* hazard ratio, *CI* confidence interval, *CKD* chronic kidney disease, *HD* hemodialysis, *COPD* chronic obstructive pulmonary disease, *CHF* congestive heart failure, *NYHA* New York Heart Association, *ACS* acute coronary syndrome, *MR* mitral regurgitation, *BITA* bilateral internal thoracic artery, *P-EF* postoperative left ventricular ejection fraction, *EF* ejection fraction

**Fig. 3:**
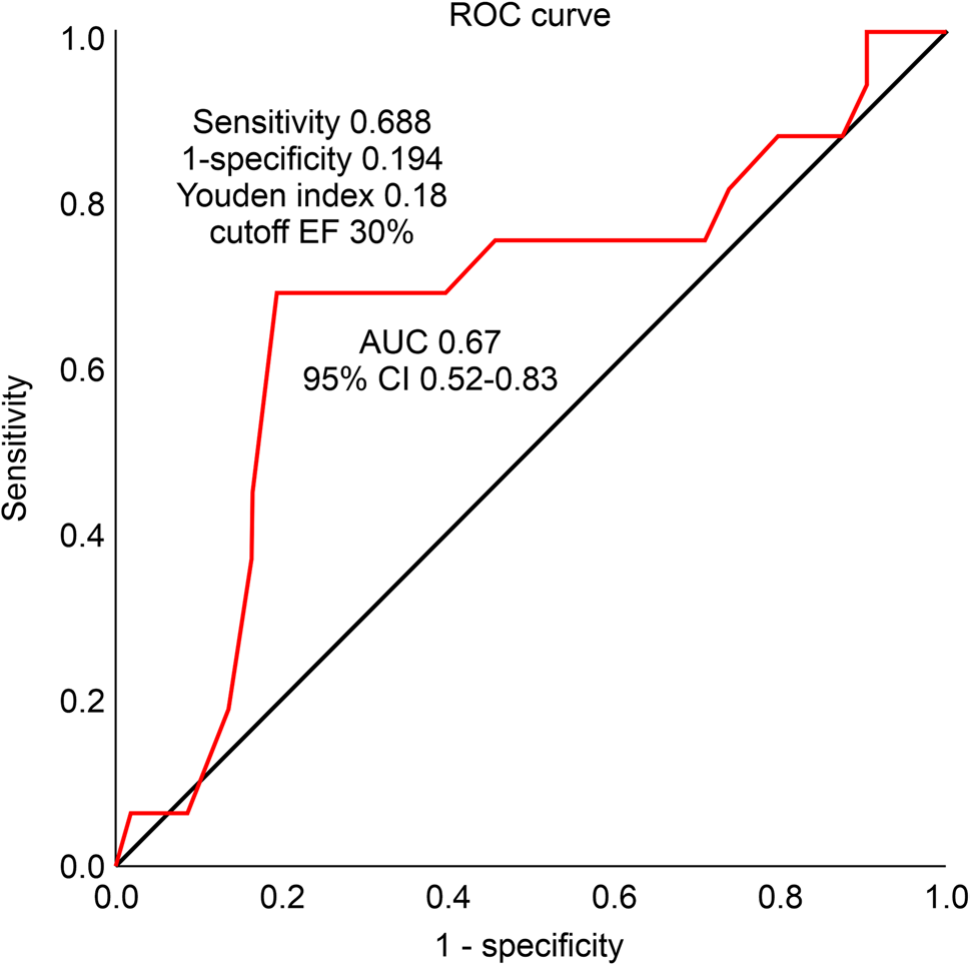
The ROC curves of postoperative EF for predicting cardiac death. The AUC was 0.67 (95%CI 0.515–0.666). The sensitivity was 69%, the specificity was 81%, and the Youden index was 18%. *ROC* receiver operation characteristic, *EF* ejection fraction, *AUC* area under curve, *CI* confidence interval

To determine the cutoff value of postoperative EF associated with long-term outcomes, we performed receiver operating characteristic (ROC) curve analysis with logistic regression. The area under the curve for long-term cardiac death was 0.67, with ROC curve analysis indicating a cutoff postoperative EF of 30% (Fig. 3). Common EF thresholds for low LV function are 30% or 35%. Accordingly, we analyzed the association of long-term outcomes with postoperative EF thresholds of 30% and 35%. There was no significant difference at 35%; however, we observed a significant difference at a postoperative EF threshold of 30%, which was also the preoperative definition of low LV function in this study (Table 3). Therefore, we classified patients with a postoperative LVEF > 30.0% and ≤ 30.0% as the responder and non-responder groups, respectively. There were only five cases with a postoperative LVEF < 25%, which was not a significant threshold.

Table 4 shows the preoperative and intraoperative characteristics of the responder and non-responder groups in the long-term group (total, 119; responder, 89; non-responder, 30). Preoperative LVEF, preoperative LVDd, and acute coronary syndrome (ACS) were significant factors.

**Table 4:** Preoperative and operative characteristics (responder versus. non-responder)

	Total (*n* = 119)	Responder (*n* = 89)	Non-responder (*n* = 30)	*P* value
Preoperative characteristics				
Age, mean ± SD y.o	66.4 ± 10.9	66.3 ± 10.2	66.4 ± 12.1	0.97
Octogenarians, *n* (%)	16(13.2)	10(11.2)	6(20.0)	0.25
Male, *n* (%)	95(75.2)	66(74.2)	24(80.0)	0.26
Hypertension, *n* (%)	84(69.4)	64(71.9)	20(66.7)	0.38
CKD on HD, n (%)	25(20.7)	21(24.0)	4(13.3)	0.12
Diabetes, *n* (%)	75(62.0)	58(65.2)	16(53.3)	0.22
Insulin dependent, *n* (%)	24(19.8)	19(21.3)	5(16.7)	0.29
COPD, *n* (%)	18(15.1)	14(15.7)	4(13.3)	0.35
CHF (NYHA ≥ 3), *n* (%)	96(80.6)	70(78.6)	26(86.7)	0.30
ACS, *n* (%)	17(14.1)	14(15.7)	1(3.3)	0.03
LVDd ± SD mm	59.0 ± 6.96	58.1 ± 6.77	61.7 ± 7.05	0.02
MR ≥ moderate, *n* (%)	14(11.7)	11(12.4)	3(10.0)	0.37
EF, mean ± SD %	24.8 ± 4.5	25.6 ± 4.24	22.0 ± 4.19	0.01
Operative characteristics				
Average distal anastomosis, Mean ± SD (range)	3.54 ± 0.94 (2–6)	3.56 ± 0.97 (2–6)	3.52 ± 0.89 (2–6)	0.84
BITA	66(55.5)	50(56.2)	16(53.3)	0.31
Emergent operation, *n* (%)	22(17.6)	19(21.3)	3(10.0)	0.08
IABP, *n* (%)	83(69.7)	59(66.3)	24(80.0)	0.08
Conversion, *n* (%)	3(2.52)	2(2.25)	1(3.33)	0.38
Complete revascularization, *n* (%)	117(98.3)	87(97.8)	30(100)	0.20

*SD* standard deviation, *CKD* chronic kidney disease, *HD* hemodialysis, *COPD* chronic obstructive pulmonary disease, *CHF* congestive heart failure, *NYHA* New York Heart Association, *ACS* acute coronary syndrome, *LVDd* left ventricular end-diastolic diameter, *MR* mitral regurgitation, *EF* ejection fraction, *BITA* bilateral internal thoracic artery, *IABP* intra-aortic balloon pumping

**Fig. 4:**
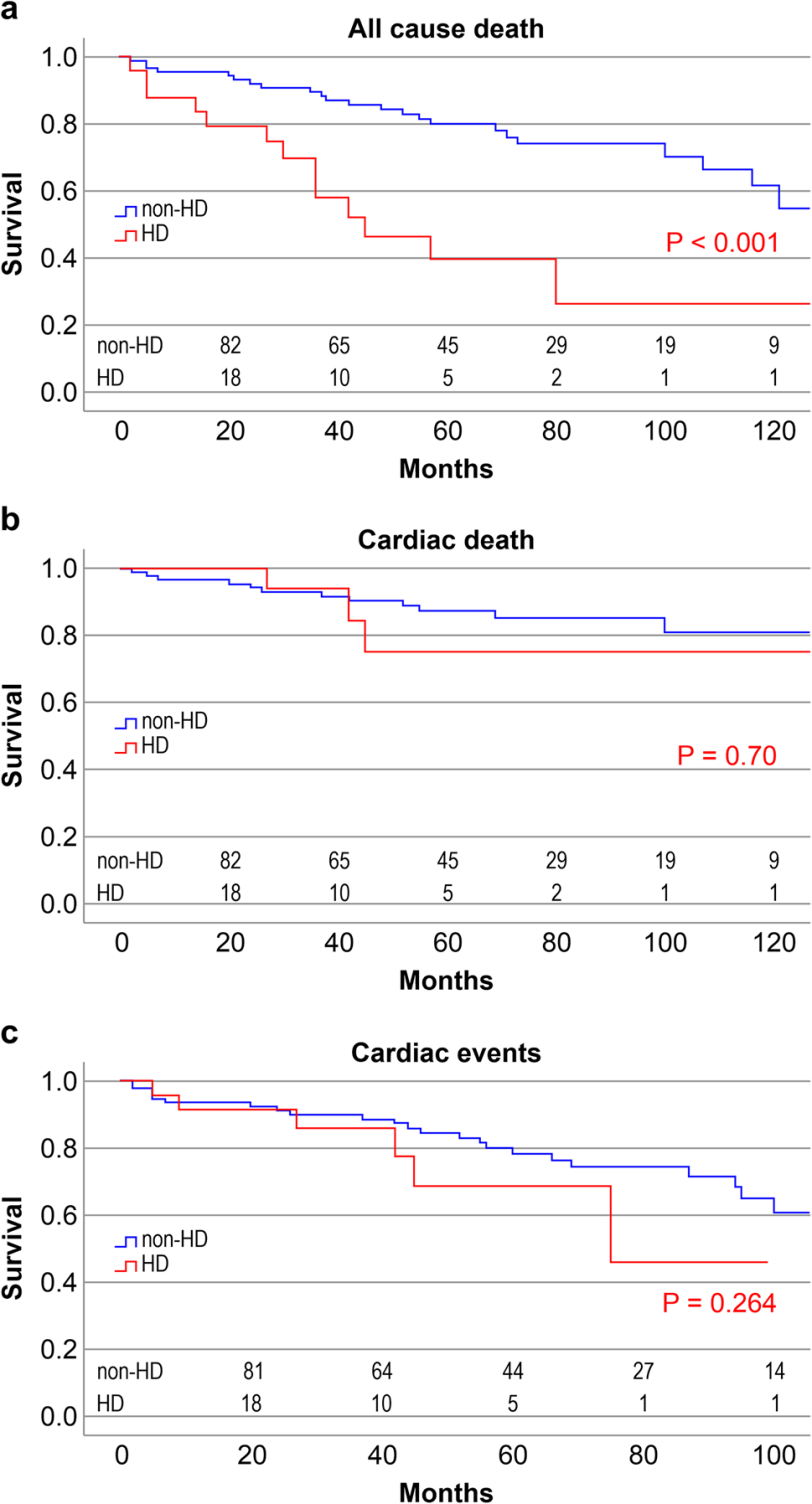
Survival analysis with Kaplan–Meier for freedom from all-cause death (**a**), cardiac death (**b**), and cardiac events (**c**) in both HD and non-HD groups. **a**. There was a significant difference between the groups (*P* < 0.001). **b**, **c**. There was no significant difference between the groups (cardiac death *P* = 0.70, cardiac events *P* = 0.26). *HD* hemodialysis

Figure 4a–c shows Kaplan–Meier survival analyses comparing patients with HD and without HD. There was a significantly higher survival rate among patients without HD than among those with HD for all-cause death (log-rank *P* < 0.001); however, there were no significant differences in the survival rates for cardiac death or cardiac events (log-rank *P* = 0.70 [cardiac death], *P* = 0.264 [cardiac events]).

**Fig. 5:**
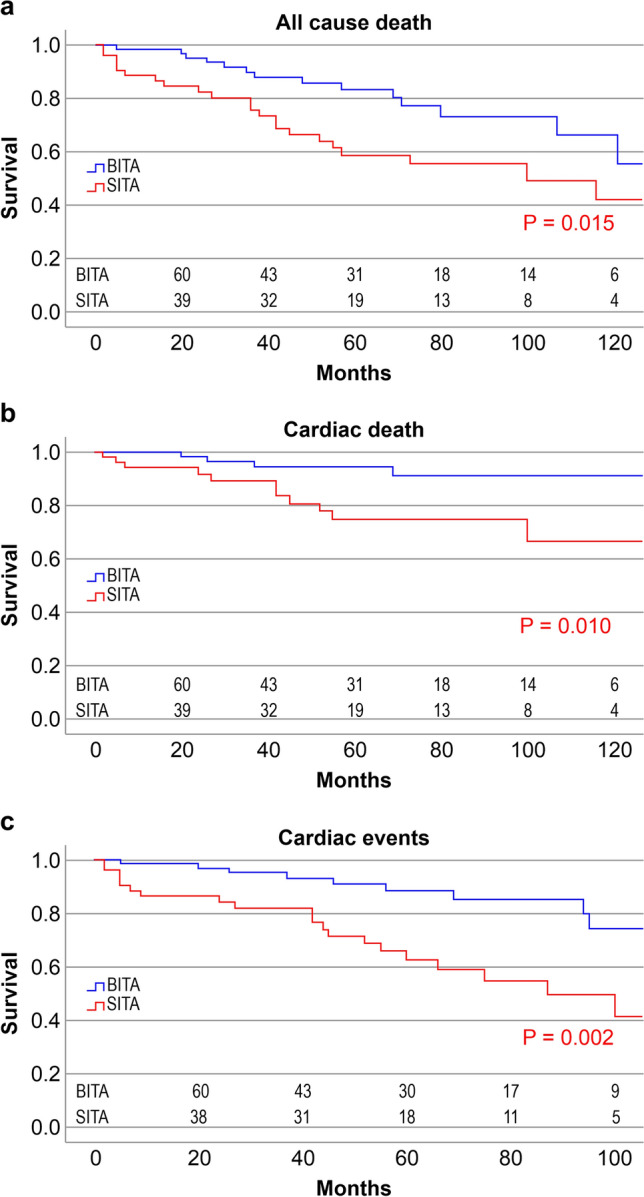
Survival analysis with Kaplan–Meier for freedom from all-cause death (**a**), cardiac death (**b**), and cardiac events (**c**) in both SITA and BITA groups. **a** There was a significant difference between the groups (*P* = 0.015). **b** There was a significant difference between the groups (*P* = 0.01). **c** There was a significant difference between the groups (*P* = 0.002). *BITA* bilateral internal thoracic artery, *SITA* single internal thoracic artery

Figure 5a–c shows Kaplan–Meier survival analyses comparing patients in the BITA and SITA groups. The freedoms from all-cause death, cardiac death, and cardiac events were significantly higher in the BITA group than in the SITA group (log-rank *P* = 0.015 [all-cause death], *P* = 0.01 [cardiac death], *P* = 0.002 [cardiac events]).

**Fig. 6:**
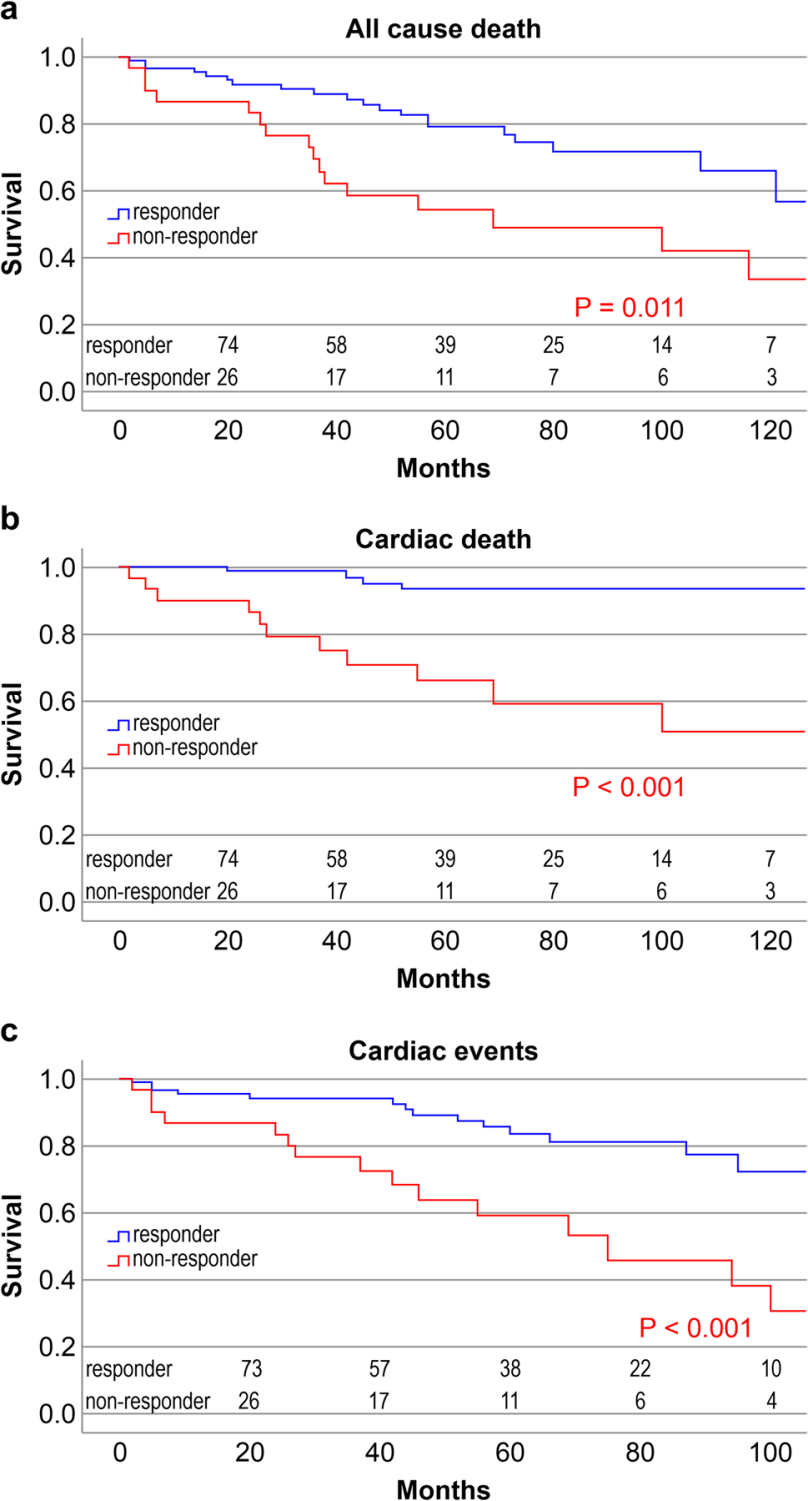
Survival analysis with Kaplan–Meier for freedom from all-cause death (**a**), cardiac death (**b**), and cardiac events (**c**) in both responder and non-responder groups. **a** There was a significant difference between the groups (*P* = 0.011). **b** There was a significant difference between the groups (*P* < 0.001). **c** There was a significant difference between the groups (*P* < 0.001)

Figure 6a–c shows Kaplan–Meier survival analyses comparing responders and non-responders. There were significantly higher survival rates for all-cause death, cardiac death, and cardiac events among responders than among non-responders (log-rank *P* = 0.011 [all-cause death], *P* < 0.001 [cardiac death and cardiac events]).

## Discussion

Our study had two important findings.

First, multivessel OPCAB for complete revascularization in patients with severe LV dysfunction was associated with acceptable in-hospital mortality and significantly improved early LV function postoperatively. Second, OPCAB with BITA grafting can provide long-term benefits in terms of cardiac death and cardiac events. However, these long-term benefits were significantly limited among patients with postoperative LVEF ≤ 30.0% and those undergoing chronic HD.

Although severe LV dysfunction is an independent risk factor for early and long-term mortality in CABG [1–4], the in-hospital mortality rate in our study was 1.64%. In the STICH trial, the 30-day postoperative mortality rate was 5.1%; in the Society of Thoracic Surgeons (STS) National Database, the in-hospital mortality rate was 3.1% after ONCAG and OPCAB; and in the Japan Adult Cardiovascular Surgery Database (JCVSD), the postoperative mortality rates were 6.1% and 3.3% after ONCAB and OPCAB, respectively [6, 7, 25]. These previous reports are consistent with our results. Compared with ONCAB, OPCAB has been reported to reduce operative mortality and early morbidities [7–9]. According to the STS National Database, OPCAB was associated with a significantly reduced adjusted risk of early morbidity and mortality in patients who underwent CABG with an EF < 0.30 [7]. However, this advantage was not maintained in the mid- or long-term [9, 10]. This may be attributed to the incomplete revascularization with fewer anastomoses occurring in the OPCAB group than in the ONCAB group [10]. Specifically, the total blood supply is significantly reduced in patients with severe LV dysfunction; therefore, a sufficient number of anastomoses are required to extensively improve myocardial ischemia. In some cases, even three anastomoses may be insufficient for complete revascularization. In our study, the average number of anastomoses was 3.54, with a complete revascularization rate of 97.0%. This was also more acceptable when compared with the number of anastomoses in previous studies: 3.00 in the STITH trial, 3.00 in the STS National Database, and 3.35 in the JCVSD [6, 7, 25]. Moreover, according to the JCVSD, OPCAB, which was performed in 57.6% of patients undergoing CABG, was associated with significantly reduced early mortality and morbidity among patients with EF < 0.30 in Japan [25, 26]. Therefore, OPCAB can be performed with an acceptable quality with respect to the number of anastomoses and rate of complete revascularization in patients with LVEF ≤ 30%.

Other benefits of OPCAB include a short ischemic time and early remodeling of LV function [27]. Letsou et al. reported short-term improvement in cardiac function after OPCAB [28]. These early improvements may contribute to early subjective clinical enhancements and maintenance of long-term benefits.

Our findings demonstrated the long-term benefits of OPCAB with BITA grafting compared with SITA grafting. However, these benefits were not observed among patients undergoing HD or non-responders with respect to all-cause death. In both groups, approximately 24.8% of patients did not show LVEF improvement; accordingly, they did not experience the long-term benefits of OPCAB with BITA grafting. Although CABG with BITA grafting has long-term benefits for patients with normal LV function [11–13, 16], some studies have reported no significant differences between the BITA and SITA groups in long-term outcomes among patients with severe LV dysfunction. This may be associated with the high rate of cardiac events resulting from LV dysfunction [11, 16, 17]. Contrastingly, Gatti et al. reported long-term survival benefits of CABG with BITA grafting for complete myocardial revascularization. However, these benefits were observed only among patients who showed substantial improvement in early LV function [29]. In our study, OPCAB with a high rate of complete revascularization showed a significant advantage in improving early LV function. This may explain the long-term benefits of OPCAB with BITA grafting. However, patients without improved early LV function after surgery did not experience these benefits, which is consistent with previous studies on CABG [16, 29].

HD is another significant factor affecting long-term survival. In our study, chronic HD was a significant predictor of all-cause death but not cardiac death and events. Specifically, chronic HD patients with severe LV dysfunction did not experience the long-term benefits observed in other patients after coronary revascularization.

In our study, both the BITA and SITA groups showed early improvements in LV function after OPCAB. Had this trend persisted over the long-term, there would have been no significant between-group difference in outcomes. However, differences in graft patency between SVG, which is a common alternative graft, and ITA may substantially influence long-term prognosis.

Compared with patients with normal LV function, patients with severe LV dysfunction typically have more calcified and narrower native coronary arteries as well as a smaller vascular bed. Consequently, they often show slower graft flow; moreover, the size discrepancy between these narrow coronary arteries and the SVG may lead to blood flow congestion within the SVG. This congestion may reduce graft patency during the early treatment course. To address this issue, it might be necessary to increase the number of SVG anastomoses through sequential bypass or address potential causes of blood flow congestion, such as valve issues in SVG [30–32].

Finally, we categorized patients with a postoperative LVEF > 30% as "responders," which was equivalent to the 30% preoperative threshold and consistent with previous reports [7, 8, 16, 25]. However, although we classified them as "responder," we hypothesized that both the surgical effects and preoperative LV function would influence the long-term postoperative outcomes. Even with a high improvement rate in EF after surgery, we considered that cases with very low preoperative EF and postoperative EF < 30% should still be classified as having low LV function. Our hypothesis was supported by the fact that lower preoperative LVEF and larger preoperative LVDd were predictors of non-responder status. The early improvement in LV function following ACS may be attributed to the recovery of hibernation (Table 4).

Our findings have several clinical implications. First, except in chronic HD cases, OPCAB using BITA grafting should target complete revascularization in patients with LV dysfunction. Second, patients without improvements in LV function should be preoperatively identified through recent advanced MRI or positron emission tomography technology [33, 34]. However, further studies are warranted to confirm our findings.

## Limitations

Our study had several limitations. First, this was a retrospective study with a small sample size, which may have been insufficient to support our conclusions. Second, the choice of BITA or SITA grafting was determined based on the coronary anatomy and surgeon preference, which may introduce some bias. Third, most patients were followed up by their individual cardiologists; accordingly, the lack of standardized medical treatment may have introduced some bias.

## Conclusion

OPCAB can be performed for patients with LV dysfunction (EF ≤ 30%). Our findings showed that OPCAB was associated with an acceptable hospital mortality and improved early LV function postoperatively. The significant predictors of long-term cardiac death and events were postoperative LV dysfunction (EF ≤ 30%) and no use of BITA grafts. Complete myocardial revascularization in OPCAB with BITA grafting can improve early postoperative LV function as well as yield long-term benefits with respect to cardiac death and events. However, these benefits are not observed among HD patients and patients without postoperative improvement in early LV function.
